# Changes in nutritional status of children who lived in temporary shelters in Bhaktapur municipality after the 2015 Nepal earthquake

**DOI:** 10.1186/s41182-020-00225-8

**Published:** 2020-06-28

**Authors:** Bhim Gopal Dhoubhadel, Ganendra Bhakta Raya, Dhruba Shrestha, Raj Kumar Shrestha, Yogendra Dhungel, Motoi Suzuki, Michio Yasunami, Chris Smith, Koya Ariyoshi, Christopher M. Parry

**Affiliations:** 1 School of Tropical Medicine and Global Health (TMGH), Nagasaki University, 1-12-4 Sakamoto, Nagasaki, 852-8523 Japan; 2 Siddhi Memorial Hospital, Bhaktapur, Nepal; 3 Institute of Tropical Medicine, Nagasaki University, Nagasaki, Japan; 4 Present address: Michio Yasunami, Life Science Institute, Saga-Ken Medical Centre Koselkan, Saga, Japan; 5 Graduate School of Biomedical Sciences, Nagasaki University, Nagasaki, Japan; 6 Clinical Sciences, Liverpool School of Tropical Medicine, Liverpool, UK; 7 Institute of Infection and Global Health, University of Liverpool, Liverpool, UK

**Keywords:** Earthquake, Malnutrition, Children, Nepal, Nutrition, Disaster, Wasting, Underweight, Stunting

## Abstract

**Background:**

The nutritional status of children may deteriorate after natural disasters such as earthquakes. A 7.8 Richter scale earthquake struck Nepal in 2015 that affected 1.1 million children. Children whose homes were destroyed and had to live in temporary shelters were at risk of malnutrition. With the support of Nagasaki University School of Tropical Medicine and Global Health (TMGH) and Siddhi Memorial Hospital (SMH), we conducted a nutritional survey of under-5 children living in temporary shelters in Bhaktapur Municipality in 2015 immediately after the earthquake and a follow-up survey in 2017.

**Results:**

We found 591 under-5 children living in 22 temporary shelters in 2015. A total of 285 children were followed up and re-assessed in 2017. In a paired analysis (*n* = 285), the prevalence of underweight children increased from 10.9% in 2015 to 14.0% in 2017 (*P* < 0.001), stunting increased from 26.7 to 31.9% (*P* = 0.07), and wasting decreased from 4.2 to 2.5% (*P* = 0.19).

**Conclusions:**

Children who lived in temporary shelters after the 2015 Nepal earthquake might be at increased risk of a deterioration in nutritional status.

## Background

In addition to human casualties, earthquakes can cause the destruction of houses and stored food, and deaths of livestock that can result in food deprivation and may lead to malnutrition among children [1]. On April 25, 2015, a 7.8 Richter scale earthquake struck Nepal. It caused more than 8800 human casualties and affected 5.6 million people, including 1.1 million children mostly in 14 districts [2]. Along with infections, malnutrition can increase the risk of child deaths. Therefore, it is essential to monitor the nutritional status of affected children after an earthquake [3].

Studies show that the prevalence of malnutrition increases after natural disasters, including earthquakes. The Wenchuan earthquake in China, a tsunami in Sri Lanka, and floods in India have been associated with a higher prevalence of malnutrition among children [4–7]. The prevalence of underweight, stunting, and wasting children rose from 0 to 5.9%, 6.6 to 10.8%, and 1.3 to 4.0%, respectively, in Kang County, China, after 2 years of the earthquake [5]. Similarly in India, the other neighbouring country of Nepal, the children were more likely to be underweight (adjusted prevalence ratio 1.86; 95% CI 1.04–2.30) and stunting (adjusted prevalence ratio 1.60; 95% CI 1.05–2.44) in flooded households than non-flooded ones [7]. Similar to the effects of these natural disasters, there could be an increased risk of malnutrition among the children after the earthquake in Nepal.

Bhaktapur was one of the 14 heavily earthquake-affected districts. It was also one of the Malnutrition and Enteric Disease (MAL-ED) study sites in Nepal. The MAL-ED study showed that the prevalence of stunting was 40% in a census survey in 2010, which was similar to the national prevalence of 41% in 2011 [8]. The National Demographic and Health Survey reported that the national average of stunting decreased to 36% in 2016 and in Bagmati province, where Bhaktapur lies, the prevalence of stunting decreased to 29% [9]. This reduction in stunting shows the general nutritional status of children in Nepal is improving over the period of recent years. However, the effects of the earthquake on nutrition of the affected children are not well studied in Nepal.

The objective of this study was to assess the changes in nutritional status of the children, who had to live in temporary shelters after the earthquake. This was important because an earthquake could increase the risk of malnutrition and it could be more severe among the children who had to live outside of their home. Therefore, immediately after the 2015 Nepal earthquake, we conducted a survey of nutrition among under-5 children in temporary shelters in Bhaktapur Municipality. We reassessed them that we were able to follow-up in 2017.

## Results

**Fig. 1:**
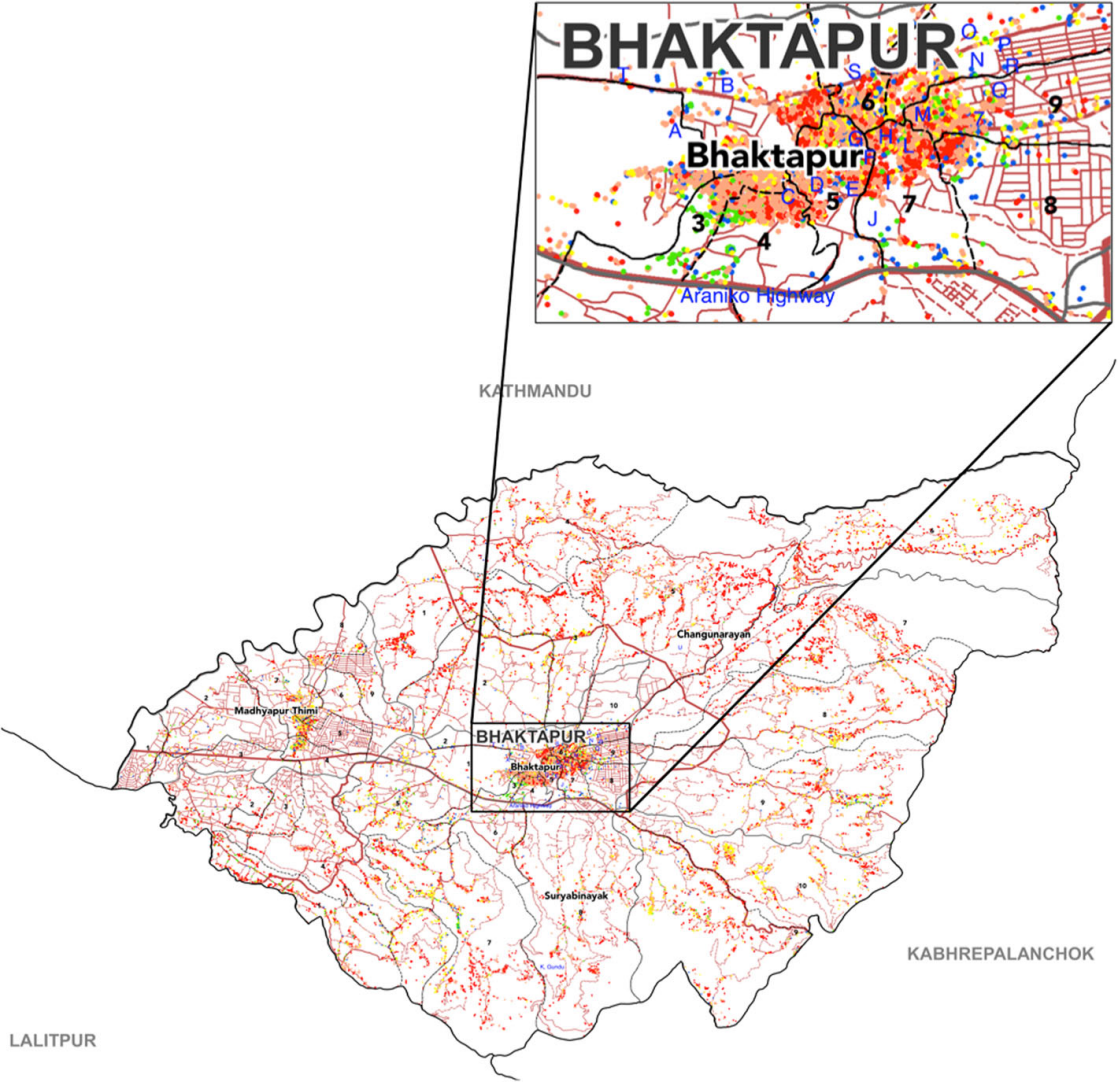
Earthquake damage map of Bhaktapur district after the 2015 Nepal earthquake. Earthquake damage in Bhaktapur district after the earthquake. The map is adapted and approved to publish from Housing Recovery and Reconstruction Platform (HRRP-Nepal) (http://hrrpnepal.org/maps/ accessed on 2018 December 20). Grades of damage are in an incremental order from 1 to 5; these are shown in green, pink, yellow, orange, and red dots, respectively. English alphabets are designed in the places where temporary shelters were situated after the 2015 Nepal earthquake

The first survey was conducted from the 19th to 26th of May 2015; we visited 22 temporary shelters in Bhaktapur Municipality. The temporary shelters and the damage due to the earthquake on the 25th of April and a major aftershock on the 12th of May are shown in Fig. 1. We assessed the nutritional status of 591 children aged between 6 and 59 months who were found living in these temporary shelters. Characteristics of the children are shown in Table 1. In the first survey, the mean (standard deviation) age of children was 32.4 (15.6) months. Newar was the main ethnic background. The nutritional characteristics of the children are shown in Table 2. Among the 591 children assessed in the first survey, 27 (4.6%) had wasting, 77 (13%) had underweight, and 163 (27.6%) had stunting (Additional file 1: Figure S1). There were six children (1%) who had severe acute malnutrition (SAM): three with mid-upper arm circumference (MUAC) less than 11.5 cm and three with severe wasting. Bilateral pedal edema was not detected in any child.

**Table 1:** Characteristics of children in the first and the follow-up surveys

Characteristics	First survey No. (%) (*n* = 591)	Follow-up survey No. (%) (*n* = 285)
Sex, female	259 (43.8)	116 (40.7)
Age groups, month		
6–12	74 (12.5)	NA
13–36	267 (45.2)	70 (24.5)
37–60	250 (42.3)	132 (46.3)
> 60	NA	83 (29.2)
Ethnicity		
Newar	413 (69.9)	232 (81.4)
Others	178 (30.1)	53 (18.6)
Permanent address		
Bhaktapur	400 (67.7)	232 (81.4)
Others	191 (32.3)	53 (18.6)
Mother’s education	NA	
University graduate		34 (11.9)
Higher secondary school		43 (15.1)
Secondary school		108 (37.9)
Literate without formal schooling		73 (25.6)
Illiterate		27 (9.5)
Type of residence	NA	
Own house		175 (61.8)
Rented house		73 (25.8)
Tent		35 (12.4)
Source of family income	NA	
Professional job		102 (35.8)
Business		63 (22.1)
Agriculture		18 (6.3)
Labourer		45 (15.8)
Others		57 (20.0)
Source of drinking water	NA	
Tap water		243 (85.3)
Others		42 (14.7)
Purification of drinking water	NA	
Filtration		151 (53.0)
Boiling		28 (9.8)
Chlorination		6 (2.1)
None		100 (35.1)
Toilet	NA	
Flush		271 (95.0)
Pit with cover		7 (2.5)
Pit without cover		7 (2.5)

Characteristics of children who were living in temporary shelters immediately after the 2015 Nepal earthquake (first survey) and after 2 years (follow-up survey) in Bhaktapur Municipality, Nepal. NA stands for not available for the data that were not collected in the first survey because it was conducted in an emergency situation just after the earthquake

**Table 2:** Characteristics of nutritional status of children in the first and the follow-up surveys

Characteristics	First survey No. (%) (*n* = 591)	First survey (paired with follow-up) No. (%) (*n* = 285)	Follow-up survey No. (%) (*n* = 285)	*P* value*
Mid-upper arm circumference (MUAC)				0.05
Red (< 115 mm)	3 (0.5)	1 (0.4)	0 (0.0)	
Yellow (115–125 mm)	18 (3.0)	7 (2.4)	2 (0.7)	
Green (> 125 mm)	570 (96.5)	277 (97.2)	283 (99.3)	
Wasting				0.19
Wasting (WHZ score ≤ 2)	27 (4.6)	12 (4.2)	7 (2.5)	
Normal	564 (95.4)	273 (95.8)	278 (97.5)	
Underweight				< 0.001
Underweight (WAZ score ≤ 2)	77 (13.0)	31 (10.9)	40 (14.0)	
Normal	514 (87.0)	254 (89.1)	245 (86.0)	
Stunting				0.07
Stunting (HAZ score ≤ 2)	163 (27.6)	76 (26.7)	91 (31.9)	
Normal	428 (72.4)	209 (73.3)	194 (68.1)	
Severe acute malnutrition (SAM)#	6 (1.0)	4 (1.4)	1 (0.4)	0.17

Comparison of nutritional status of children living in temporary shelters immediately after the 2015 Nepal earthquake (first survey) and after 2 years (follow-up survey) in Bhaktapur Municipality, Nepal. #SAM includes MUAC less than 115 mm and/or severe wasting (WHZ score ≤ 3), and/or bilateral pedal edema

*P values were calculated for pair-wise comparison for 285 children

A total of 285 (54.9%) of the children from the first survey could be traced during the follow-up survey. In 2017, 35 (12.4%) children were still living in the temporary shelters whereas 175 (61.8%) had returned to their own new home and 73 (25.8%) were living in rented housing (Table 1). The mean (standard deviation) age of children was 54.7 (17.8) months in the follow-up survey. The children who could not be followed-up (lost in follow-up) tended to be older than those who could be followed-up (mean age 34.1 vs 30.5 months; *P* = 0.004). The proportions of children with non-Newar ethnicity (40.9% among lost in follow-up vs 18.6% among followed-up; *P* < 0.001) and residence outside of the Kathmandu valley (41.8% among lost in follow-up vs 22.1% among followed-up; *P* < 0.001) were higher among lost in follow-up than among followed-up children (Additional file 2: Table S1). These data suggested that the people outside of Bhaktapur, probably from the surrounding earthquake affected hilly districts, came to the temporary shelters immediately after the earthquake, but later, they might have returned back home or gone to a different place. Additional file 3: Figure S2 shows the distribution of *z*-scores of weight-for-height (WHZ), weight-for-age (WAZ), and height-for-age (HAZ) for these two groups of children at the baseline of nutritional status in 2015. It shows there were no significant differences between the two groups of the children in terms of anthropometric parameters.

The number of children and their nutritional status in the first and the follow-up surveys are shown in Additional file 1: Figure S1. The paired comparisons of the 285 children who were assessed in the first and the follow-up surveys showed the proportion of underweight children increased from 10.9 to 14.0% (*P* < 0.001), stunting increased from 26.7 to 31.9% (*P* = 0.07), and wasting decreased from 4.2 to 2.5% (*P* = 0.19) (Table 2). SAM was detected in only one child in the follow-up survey and it was due to severe wasting.

**Fig. 2:**
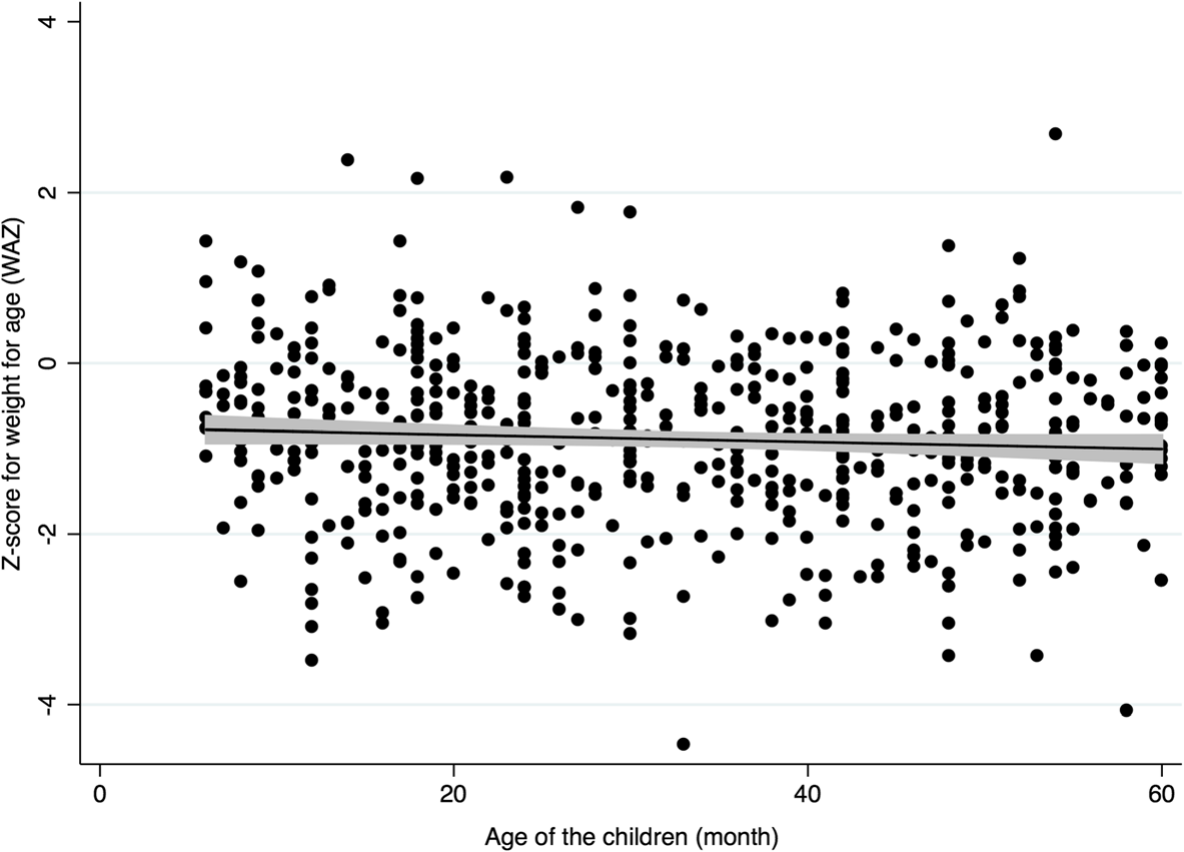
Correlation of underweight (weight-for-age *z*-scores) with the age of the children in the first survey. This scatter diagram shows the distribution of *z*-scores of weight-for-age (WAZ) that is the measure of underweight, with the fitting line and shaded 95% confidence interval, of the children living in temporary shelters in Bhaktapur Municipality immediately after the 2015 Nepal earthquake (*n* = 591, *P* = 0.108)

**Fig. 3:**
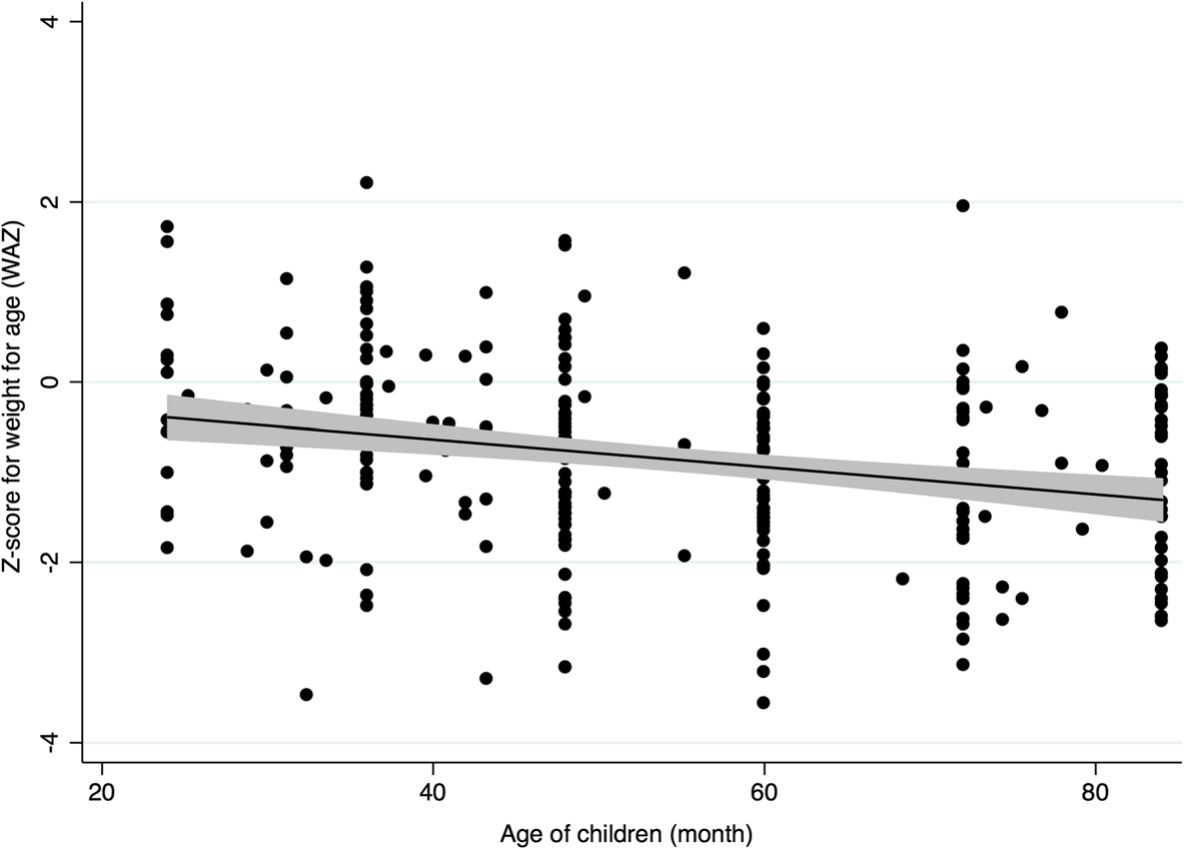
Correlation of underweight (weight-for-age *z*-scores) with the age of the children in the follow-up survey. This scatter diagram shows the distribution of *z*-scores of weight-for-age (WAZ) that is the measure of underweight, with the fitting line and shaded 95% confidence interval, of the children who lived in temporary shelters after the 2015 Nepal earthquake in Bhaktapur, Nepal, in the follow-up survey after 2 years (*n* = 285, *P* < 0.001)

We found negative correlations of the *z*-score related to wasting and underweight with the age of the children in the follow-up survey (Table 3). The correlation was more pronounced in underweight than wasting. The relationship of underweight with the age of the children in the first and the follow-up surveys are shown in Figs. 2 and 3, respectively.

**Table 3:** Correlation of nutritional status with age of the children

*Z* scores	First survey		Follow-up survey	
	Spearman’s rho	*P* value	Spearman’s rho	*P* value
Wasting (WHZ)				
All	− 0.039	0.33	− 0.188	< 0.01
Male	− 0.018	0.73	− 0.226	< 0.01
Female	− 0.074	0.23	− 0.140	0.13
Underweight (WAZ)				
All	− 0.064	0.11	− 0.255	< 0.01
Male	− 0.015	0.77	− 0.218	< 0.01
Female	− 0.121	0.05	− 0.304	< 0.01
Stunting (HAZ)				
All	− 0.001	0.96	− 0.096	0.10
Male	0.046	0.39	− 0.019	0.80
Female	− 0.063	0.30	− 0.198	0.03

Correlations of *z*-scores of weight-for-height (WHZ), weight-for-age (WAZ), and height-for-age (HAZ) with age of the children living in temporary shelters immediately after the 2015 Nepal earthquake (first survey) and at the follow-up after 2 years (follow-up survey) in Bhaktapur Municipality, Nepal

## Discussion

This study shows the changes in nutritional status of the children who lived in temporary shelters after the earthquake in Bhaktapur Municipality, Nepal in 2015. There was no increase in proportion of wasting and SAM in 2017; however, the proportion of underweight increased by 3.1% and underweight tended to be correlated with the age of the children after 2 years following the earthquake.

Immediately after the earthquake, the Government of Nepal activated the humanitarian clusters recommended by the United Nations. The Nutrition Cluster led by the Ministry of Health and Population, which included 28 national and international agencies, implemented programmes to prevent deterioration of nutritional status of children [10]. Some of the programmes were promotion of breast-feeding and distribution of multiple micronutrient powders for home fortification of food to earthquake-affected children aged from 6 to 23 months. Similarly, the Food Security Cluster, working closely with the Ministry of Agricultural Development, provided assistance through food distributions, conditional cash transfers, and cash-for-work programmes. It seems that these programmes had some positive effects on the nutritional status of the children. SAM was found in six (1%) of the 519 children in 2015 and only one (0.4%) of the 285 children in 2017. Among the children assessed in the follow-up survey, the proportion of wasting had decreased from 4.6 to 2.5%. The prevalence of wasting of 2.5% in 2017 was lower than that of 4.2% in a survey conducted in this province in 2016 [9]. Our findings of decrease in wasting is also similar to a household survey that reports the prevalence of wasting, 4.5% in 2014 and 2.1% in 2016 in seven earthquake-affected areas in Nepal [11].

We found the proportion of underweight children increased from 10.9% in 2015 to 14.0% in 2017 and stunting rose from 26.7 to 31.9% over the period. These figures are comparable with the data of the Nepal Demographic and Health Survey 2016, which shows the proportion of underweight children was 13.3% and stunting was 29.4% in Bagmati province, where Bhaktapur Municipality lies [9]. We found a higher proportion of children with stunting (26.7% in 2015 and 31.9% in 2017) than the report that analyses the nutritional status of children in 2014 (23.1%) and 2016 (21.6%) in seven earthquake-affected areas [11]. We also found that being underweight was correlated with the age of the children in 2017. The increase in the proportion of underweight children in 2017 may be due to various reasons, including the inadequacy of balance diet, psychological trauma due to loss of home, and repeated infections in temporary shelters due to low level of hygiene and overcrowding.

Food supplementation was shown to help reduce malnutrition among children after earthquakes in Haiti and India [12, 13]. However, after the Wenchuan Earthquake in China, the nutritional status of children deteriorated despite the nutritional support [4]. Another study showed micronutrient deficiencies of iron, zinc, vitamin A, and vitamin B12 and a high prevalence of stunting in the earthquake affected regions in China [14]. Two years after the Wenchuan earthquake, the proportions of wasting, underweight, and stunting were found significantly high in the children even though the government provided a food basket of 1730 Kcal/day after the earthquake [4]. Children’s physiological requirement of fat and micronutrients might not have met by the food basket, which mainly contained basic energy giving food (cereals and wheat flour). Children who survive an earthquake may also suffer from the emotional, physical, and social deprivation that may also affect their normal growth [15]. Thus, they also need psychological, emotional, and social support in addition to a balance diet that provides enough energy, micronutrients, and vitamins**.**

These surveys in Bhaktapur, Nepal, along with other research findings mentioned above, show that the nutritional status of children is at risk of deterioration after an earthquake. Distribution of food alone seems not adequate to prevent malnutrition in these children. Studies on risk factors for deterioration of nutritional status that include environmental, psychosocial, and economic aspects are needed to understand the drivers of malnutrition after an earthquake.

These surveys had limitations. In the first survey, we could not ask detailed demographic characteristics, and in both surveys, we could not assess anaemia and micronutrient status, psychological aspects, and environmental factors that could increase the risk of malnutrition. Therefore, this study could not show a complete picture of various factors associated with malnutrition. Almost half of the children could not be traced in the follow-up survey, which led to the lower number of children for the comparison as well as various potential biases. We tried to mitigate this by the paired comparison in data analysis. By comparing whether there were any significant differences in nutritional status of children between those who could be followed up and not, we tried to see whether these two groups of children were different from the beginning.

## Conclusion

This study suggests that 2015 Nepal earthquake had some negative effects on the growth of the children who were living in temporary shelters. Underweight and stunting increased after 2 years. The children in temporary shelters need close observation for normal growth and may need public health programmes that can address the psychosocial and emotional needs of the children in addition to nutritional support to decrease the long-term effects of the earthquake.

## Methods

SMH is the only children’s hospital in Bhaktapur, Nepal. Immediately after the 2015 Nepal earthquake, with the support from TMGH and SMH, we surveyed nutritional status of under-5 children in 22 temporary shelters in Bhaktapur Municipality in May 2015. The temporary shelters in Bhaktapur Municipality are shown in Fig. 1.

A team of three medical doctors, three nurses, and one nutritionist visited all the temporary shelters in the municipality. Data were collected using a questionnaire for demographic information (age, sex, address, etc.) and trained nurses took anthropometric measurements (weight, height, mid-upper arm circumference, and pedal oedema, etc.). Weight was measured to the nearest 100 g using a digital scale (SECA). Supine length was measured to the nearest 0.1 cm for babies less than 24 months old and standing height for children 24 to 59 months old by using a portable stadiometer (SECA). Disposable MUAC tapes from the United Nations Children’s Fund (UNICEF) were used to measure MUAC.

We followed-up the children from the first survey after 2 years of the earthquake in 2017. We contacted the families using mobile phone numbers recorded in the first survey. In the follow-up survey, we collected more information: mother’s education, type of present residence, source of family income, source of drinking water, and type of toilet use, etc. In both surveys, severely malnourished children and children who needed medical treatment were referred to SMH for free investigations and treatment.

Data were collected onto a paper questionnaire and later transferred to an electronic database using Epi Info software from the Centers for Disease Control and Prevention, USA in the research facilities in SMH. Wasting, underweight, and stunting were defined according to UNICEF definitions [16]. Wasting was defined as WHZ *z*-score less than − 2, underweight as WAZ *z*-score less than − 2, and stunting as HAZ *z*-score less than − 2. These three nutritional parameters are defined as severe once their respective *z-*score is less than − 3. SAM was defined as severe wasting (WHZ *z-*score less than − 3) or MUAC less than 115 mm or in the presence of bilateral pedal edema [17]. *Z*-score was calculated by using Emergency Nutrition Assessment 2011 software by Action Against Hunger Canada (Toronto, Canada). Correlation of *z*-score with age was determined by Spearman’s test. Statistical analysis was performed using Stata 14 (StataCorp, Texas, USA).

## Supplementary information

**Additional file 1: Figure S1**. Changes of nutritional status of children from the first survey to the follow-up survey. Flowchart showing the number of children screened and their nutritional status in the first survey, and the changes in the status in the follow-up survey.

**Additional file 2: Table S1**. Characteristics of children who could be followed-up and who could not be followed-up.

**Additional file 3: Figure S2**. Comparisons of nutritional status of the children who could be followed-up and who could not be followed-up. The kernel-density plots show the distributions of *z*-scores of weight-for-height (WHZ), weight-for-age (WAZ), and height-for-age (HAZ) that are the measures of wasting, underweight, and stunting, respectively, of the children who could be followed-up (*n* = 285) and who could not be followed-up (*n* = 306).

## Data Availability

De-identified data of this study are available upon reasonable request from the corresponding author.

## Abbreviations

HAZ: Height-for-age
Mal-Ed: Malnutrition and Enteric Disease
MUAC: Mid upper-arm circumference
SAM: Severe acute malnutrition
SMH: Siddhi Memorial Hospital
TMGH: School of Tropical Medicine and Global Health
UNICEF: The United Nations Children’s Fund
WAZ: Weight-for-age
WHZ: Weight-for-height
