# Correction to: Changes in nutritional status of children who lived in temporary shelters in Bhaktapur municipality after the 2015 Nepal earthquake

**DOI:** 10.1186/s41182-020-00269-w

**Published:** 2020-09-22

**Authors:** Bhim Gopal Dhoubhadel, Ganendra Bhakta Raya, Dhruba Shrestha, Raj Kumar Shrestha, Yogendra Dhungel, Motoi Suzuki, Michio Yasunami, Chris Smith, Koya Ariyoshi, Christopher M. Parry

**Affiliations:** 1 School of Tropical Medicine and Global Health (TMGH), Nagasaki University, 1–12-4 Sakamoto, Nagasaki, 852–8523 Japan; 2 Institute of Tropical Medicine, Nagasaki University, 1–12-4 Sakamoto, Nagasaki, 852–8523 Japan; 3 Siddhi Memorial Hospital, Bhaktapur, Nepal; 4 Present address: Michio Yasunami, Life Science Institute, Saga-Ken Medical Centre Koselkan, Saga, Japan; 5 Graduate School of Biomedical Sciences, Nagasaki University, Nagasaki, Japan; 6 Clinical Sciences, Liverpool School of Tropical Medicine, Liverpool, UK; 7 Institute of Infection and Global Health, University of Liverpool, Liverpool, UK

**Correction to: Trop Med Health 48, 53 (2020)**

**https://doi.org/10.1186/s41182-020-00225-8**

Following publication of the original article [1], the authors would like to add affiliation 2 into Bhim Gopal Dhoubhadel’s affiliations: Bhim Gopal Dhoubhadel 1,2. In addition, the full address of the affiliation 2 is changed as below.

**Institute of Tropical Medicine, Nagasaki University, 1–12-4 Sakamoto, Nagasaki, 852–8523, Japan**
