# Supplementary figures and images for: Changes in nutritional status of children who lived in temporary shelters in Bhaktapur municipality after the 2015 Nepal earthquake
Source: Trop Med Health. 2020 Jun 28;48:53. doi: 10.1186/s41182-020-00225-8 (PMC7321544; doi:10.1186/s41182-020-00225-8)

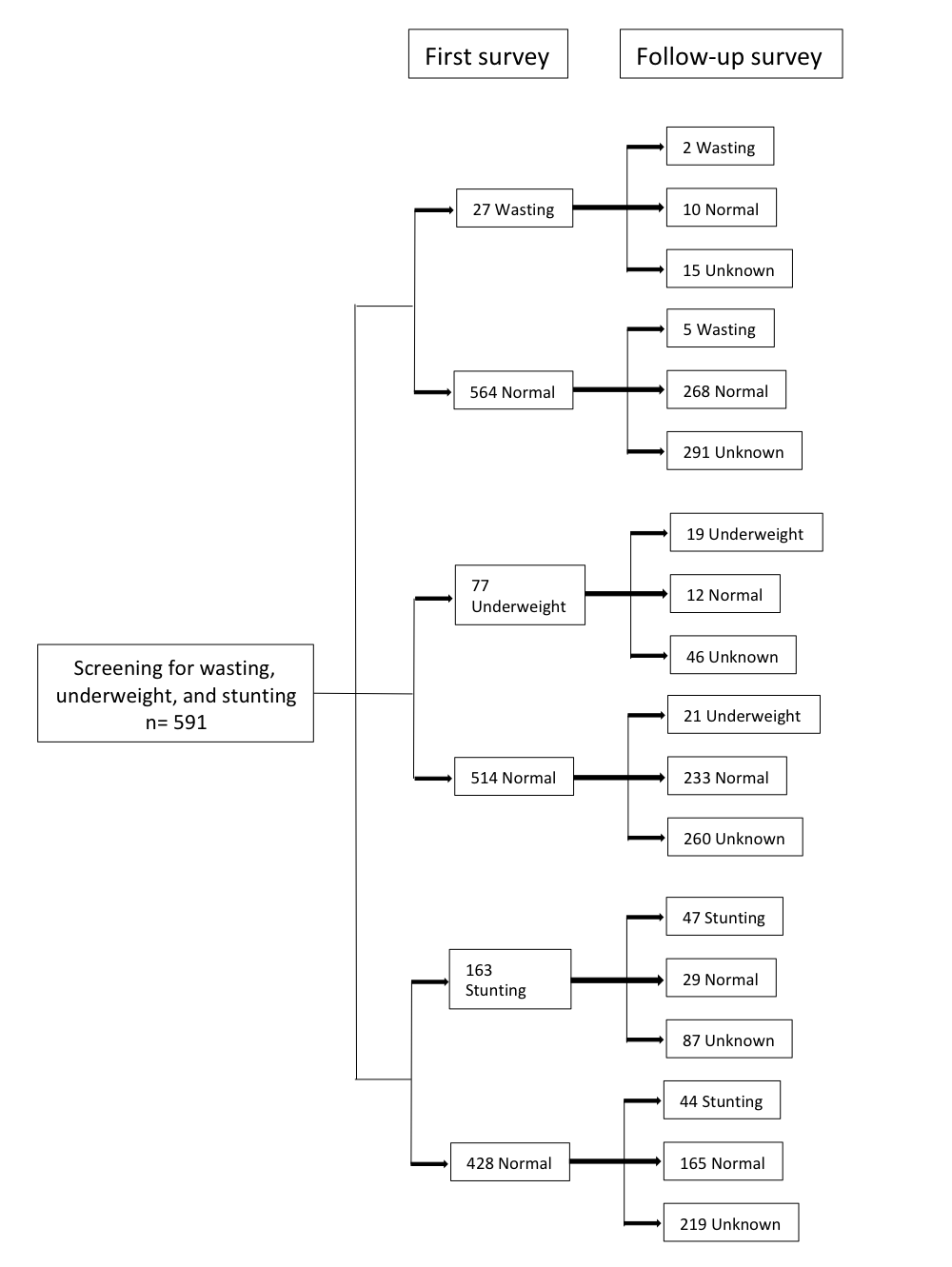

Supplement: Supplementary file 1 — Additional file 1: Figure S1. Changes of nutritional status of children from the first survey to the follow-up survey. Flowchart showing the number of children screened and their nutritional status in the first survey, and the changes in the status in the follow-up survey. [file 41182_2020_225_MOESM1_ESM.tif]

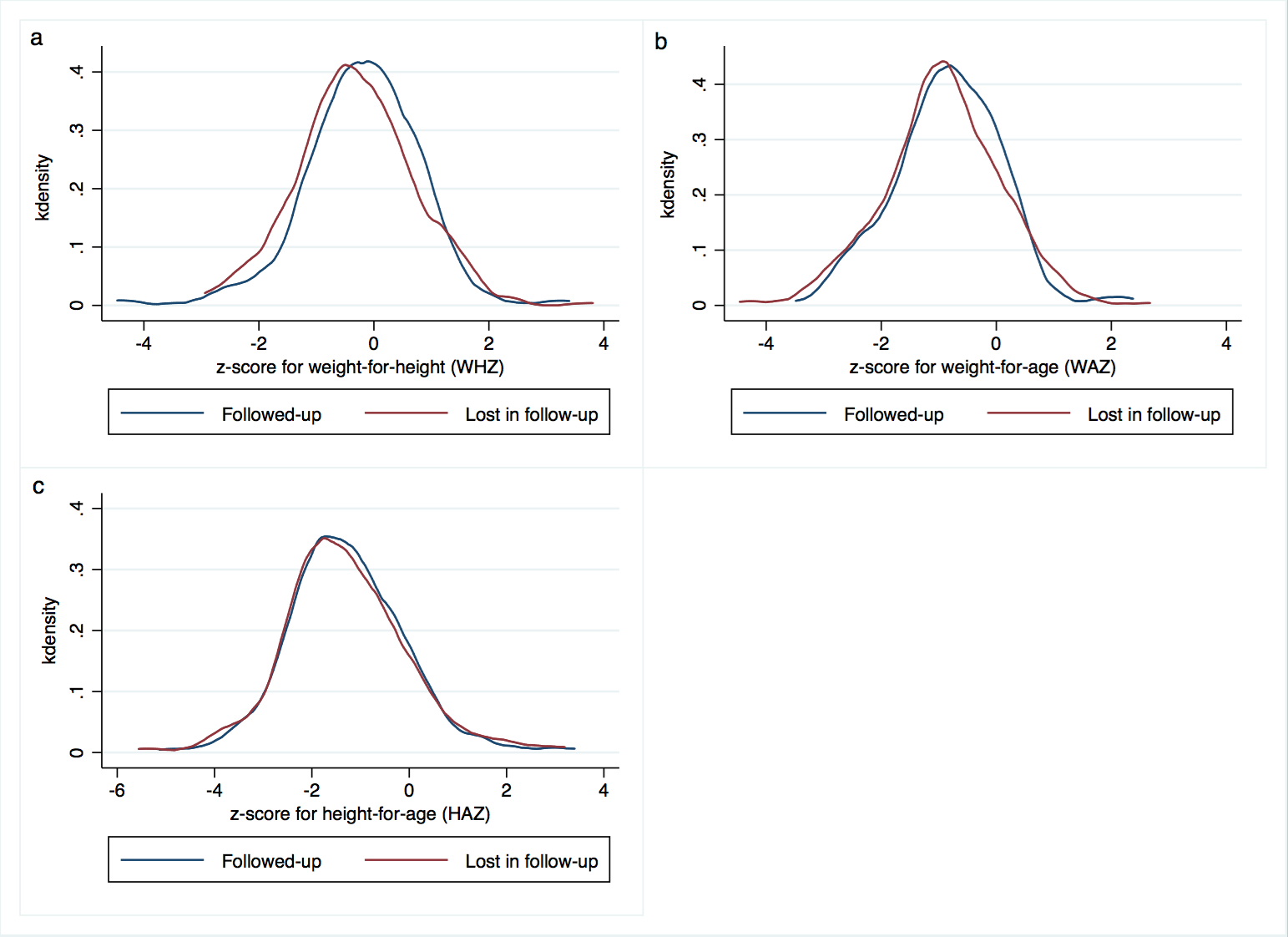

Supplement: Supplementary file 3 — Additional file 3: Figure S2. Comparisons of nutritional status of the children who could be followed-up and who could not be followed-up. The kernel-density plots show the distributions of z-scores of weight-for-height (WHZ), weight-for-age (WAZ), and height-for-age (HAZ) that are the measures of wasting, underweight, and stunting, respectively, of the children who could be followed-up (n = 285) and who could not be followed-up (n = 306). [file 41182_2020_225_MOESM3_ESM.tif]
