# Supplementary material for: Changes in nutritional status of children who lived in temporary shelters in Bhaktapur municipality after the 2015 Nepal earthquake
Source: Trop Med Health. 2020 Jun 28;48:53. doi: 10.1186/s41182-020-00225-8 (PMC7321544; doi:10.1186/s41182-020-00225-8)
Supplement: Supplementary file 2 — Additional file 2: Table S1. Characteristics of children who could be followed-up and who could not be followed-up. [file 41182_2020_225_MOESM2_ESM.docx]

Supplementary Table 1. Characteristics of children who could be followed-up and who could not be followed-up

| Characteristics | First survey  No. (%)  (n= 591) | Base line characteristics | | *P*-value |
| --- | --- | --- | --- | --- |
|  |  | Followed-up  No. (%)  (n=285) | Could not be followed-up  No. (%)  (n=306) |  |
| Sex, female | 259 (43.8) | 116 (40.7) | 143 (46.7) | 0.14 |
| Age, mean (month) | 32.4 | 30.5 | 34.1 | 0.004 |
| Age groups (month)  6-12  13-36  37-60  >60 | 74 (12.5)  267 (45.2)  250 (42.3)  NA | 40 (14.0)  136 (47.7)  109 (38.3) | 34 (11.1)  131 (42.8)  141 (46.1) | 0.14 |
| Ethnicity  Newar  Others | 413 (69.9)  178 (30.1) | 232 (81.4)  53 (18.6) | 181 (59.1)  125 (40.9) | <0.001 |
| Permanent address  Bhaktapur  Others | 400 (67.7)  191 (32.3) | 222 (77.9)  63 (22.1) | 178 (58.2)  128 (41.8) | <0.001 |
| Mid-upper arm circumference (MUAC)  Red (<115 mm)  Yellow (115-125 mm)  Green (>125 mm) | 3 (0.5)  18 (3.0)  570 (96.5) | 1 (0.4)  7 (2.4)  277 (97.2) | 2 (0.7)  11 (3.6)  293 (95.7) | 0.62 |
| Wasting  Wasting (WHZ score <-2)  Normal | 27 (4.6)  564 (95.4) | 12 (4.2)  273 (95.8) | 15 (4.9)  291 (95.1) | 0.68 |
| Underweight  Underweight (WAZ score <-2)  Normal | 77 (13.0)  514 (87.0) | 31 (10.9)  254 (89.1) | 46 (15.0)  260 (85.0) | 0.13 |
| Stunting  Stunting (HAZ score <-2)  Normal | 163 (27.6)  428 (72.4) | 76 (26.7)  209 (73.3) | 87 (28.4)  219 (71.6) | 0.63 |
| Severe acute malnutrition (SAM) | 6 (1.0) | 4 (1.4) | 2 (0.7) | 0.43* |

Comparison of general characteristics and nutritional status of children living in temporary shelters after the 2015 Nepal Earthquake (first survey) who could be followed-up (n=285) and those who could not be followed-up (n=306) in Bhaktapur Municipality, Nepal.

* Fisher's exact test
